# Association between endometrial cancer and subsequent risk of fracture: a national cohort study

**DOI:** 10.3389/fendo.2025.1570426

**Published:** 2025-10-07

**Authors:** Wen-Hsuan Tsai, Min-Shu Hsu, Chia-Sui Weng, Hsin-Yin Hsu, Cheng-Tzu Hsieh, Tzu-Lin Yeh, Kuo-Liong Chien, Chun-Chuan Lee, Ming-Nan Chien, Ming-Chieh Tsai

**Affiliations:** ^1^ Division of Endocrinology and Metabolism, Department of Internal Medicine, MacKay Memorial Hospital, Taipei, Taiwan; ^2^ Department of Medical Research, MacKay Memorial Hospital, New Taipei, Taiwan; ^3^ Department of Gynecology and Obstetrics, MacKay Memorial Hospital, Taipei, Taiwan; ^4^ Institute of Epidemiology and Preventive Medicine, College of Public Health, National Taiwan University, Taipei, Taiwan; ^5^ Department of Medicine, MacKay Medical Collage, New Taipei, Taiwan; ^6^ Department of Family Medicine, Taipei MacKay Memorial Hospital, Taipei, Taiwan; ^7^ Department of Epidemiology, University of California, Los Angeles, Los Angeles, CA, United States; ^8^ Department of Family Medicine, Hsinchu MacKay Memorial Hospital, Hsinchu, Taiwan; ^9^ Department of Internal Medicine, National Taiwan University Hospital, Taipei, Taiwan; ^10^ Population Health Research Center, National Taiwan University, Taipei, Taiwan

**Keywords:** endometrial cancer, diabetes, hip fracture, vertebral fracture, extremity fracture

## Abstract

**Aims:**

Most endometrial cancer (EC) cases are estrogen-dependent, and some are associated with diabetes mellitus (DM). We aimed to estimate the risk of fracture among patients with EC and those with DM.

**Materials and methods:**

A total of 20814 patients with EC were identified from the Taiwan National Cancer Registry from 2007 to 2018, with the outcome ascertainment using the National Health Insurance Research Database from 2004 to 2019. This observational study investigated the hazard ratios (HRs) for fracture and mortality events using Cox proportional hazards regression, with 95% confidence intervals (CIs). We adjusted baseline comorbidities, cancer therapy, cancer staging and grade, and pathological status of estrogen receptor and progesterone receptor. Considering the competing death events, we estimated the subdistribution hazard model to predict the probability of the fracture risk in the competing risks context.

**Results:**

Among 15,505 EC patients, there were 3,044 patients with and 12,461 patients without DM. Patients with EC exhibited a no significant association of fracture when compared to the matched general population. EC patients with DM, compared to those without DM, had a significantly increased odds of osteoporotic fracture (HR 1.29 [95% CI 1.08–1.55]), hip fracture (HR 2.37 [95% CI 1.44–3.92]), and vertebral fracture (HR 1.71 [95% CI 1.06–2.74]). Patients with DM had a no significant association of upper extremity fracture (HR 1.33 [95% CI 0.95–1.87]) compared with those with EC but without DM.

**Conclusions:**

EC patients had a no significant association of fracture, while DM increased the fracture risk in EC patients.

## Introduction

With the recent rise in incidence, endometrial cancer (EC) has become the leading gynecological cancer in developed countries (1). According to the notable symptoms, most patients are diagnosed at an early stage, accounting for 70% of endometrial cancer (2). Despite the relatively favorable prognosis, with a 5-year overall survival of 77% (3, 4), 25% of patients die for recurrent endometrial cancer within 5 years since diagnosis (2). Since most patients with EC will be long-term survivors, whether these patients will have different comorbidities from those without EC is a question of particular interest.

Bone loss has been associated with various cancer therapies (5, 6). Previous research on the lumbar spine bone mineral density (BMD) in patients with gynecological cancer demonstrated a positive correlation with body mass index (BMI) (7), but a negative correlation with the age of patients (7). As most EC cases are estrogen-dependent, a rapid decrease in estrogen may cause abrupt changes in BMD. This has been proven by the negative correlation between EC and BMD (7). Diabetes mellitus (DM), as a common comorbidity of EC, is not only a poor prognostic factor for EC, but also a risk factor for the development of EC (8). The underlying mechanism includes obesity, insulin resistance and chronic inflammation, hyperinsulinemia, hyperglycemia and epithelial-mesenchymal transition (EMT) (8). Despite greater BMD among patients with DM than those without DM, the higher risk of fracture in patients with DM may be due to inappropriate distribution of bone mass, deterioration in bone microarchitecture owing to the reduction of bone strength and disturbed repairment and adaptation of bone mechanisms (9, 10). In addition, several studies have reported that older adults with DM have a higher risk of falls when compared to those without DM (11, 12), which may also increase risk of fracture in patients with DM.

Our aim is to evaluate the fracture risk among patients with EC comparing with the general population, and whether EC patients with DM may associate an increased fracture risk when compared with those without DM.

## Methods and materials

### Data source

The National Taiwan Cancer Registry Database (NTCRD) is a nationally representative cancer dataset based on a 97.8% representative sample of Taiwanese cancer patients, which was designed and administrated by Ministry of Health and Welfare in 1979. Based on the Taiwan National Health Insurance program, which has covered 99.6% of Taiwanese residents under the national healthcare policy since 1995, our study utilized the Taiwan National Health Insurance Research Database (NHIRD). A total of 20814 patients with EC were identified from the NTCRD from January 1, 2007 to December 31, 2018. The demographics ascertained from the NHIRD included participants’ basic characteristics, household economic status, occupation categories, and medical records, encompassing diagnoses, medication prescriptions, and procedures from outpatient clinics, inpatient departments, and emergency departments, covering the period from January 1, 2004, to December 31, 2019. All individuals in both the NTCRD and NHIRD were de-identified and did not require participant consent. This study complied with the Declaration of Helsinki and was reviewed by the Ethics Committee of Mackay Memorial Hospital (21MMHIS398e).

### Study design

**Figure 1:**
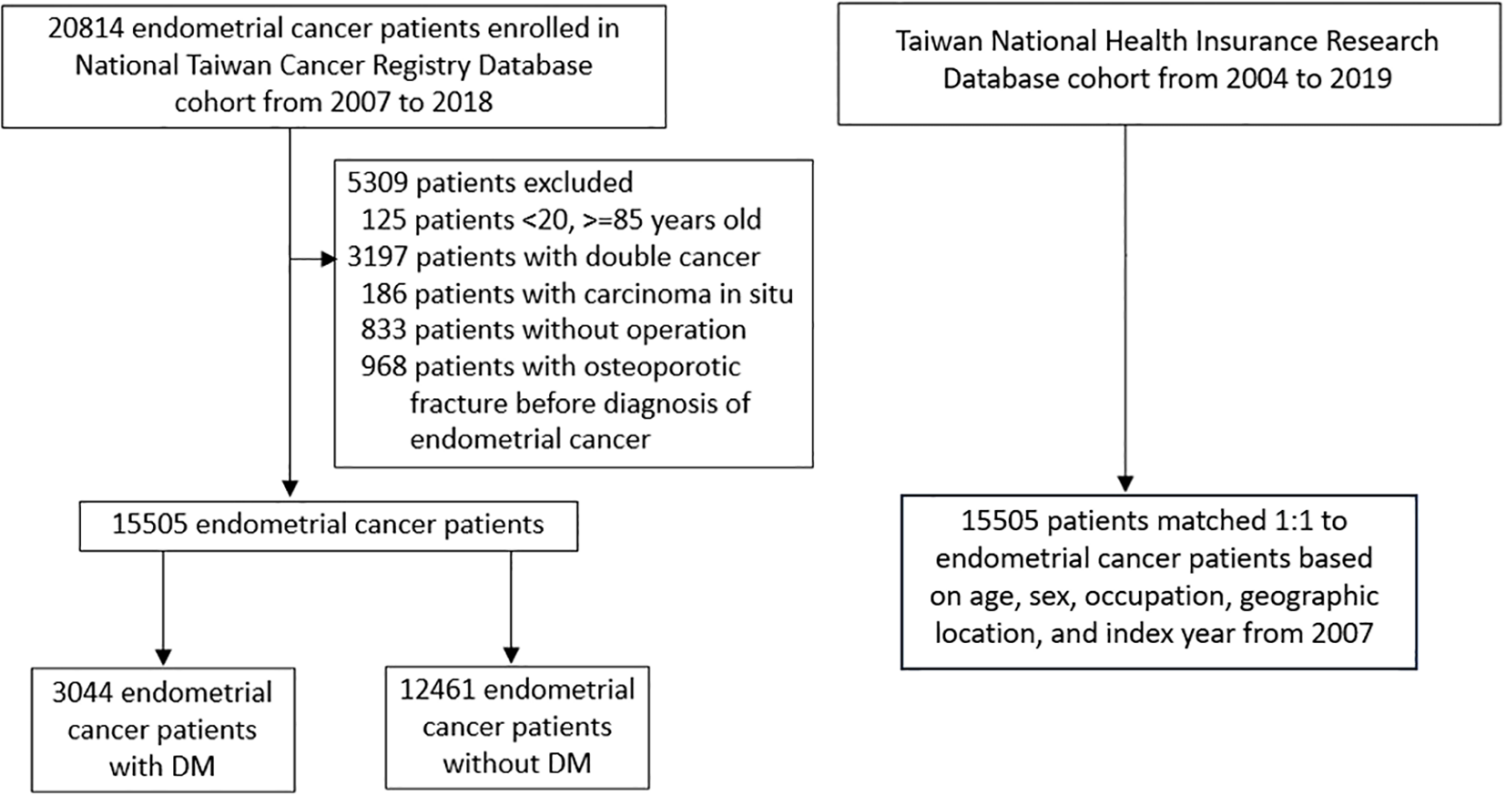
Flow chart.

First, we identified 15,505 patients with EC (2007–2013 ICD-O-FT: T-182; 2013–2018 ICD-O-3: C54.0, C54.1, C54.3, C54.8, and C54.9) who were 20 years of age or older between 2007 and 2018 (**Figure 1**). We excluded patients with a history of cancer diagnosed before 2007, those with double cancer, and those with fractures before the index date. Because surveillance of osteoporosis is a self-pay examination in Taiwan, only a small proportion of population have received osteoporosis examination. Hence, the ICD coding of osteoporosis may underestimate the accurate proportion of osteoporosis. In addition, since osteoporosis medications are only reimbursed by health insurance with strict criteria, many patients have to use osteoporosis medication by self-pay, which could not be evaluated by NHIRD. Therefore, we only excluded patients with history of osteoporotic fractures. These patients were further stratified into two groups: 3,044 patients with and 12,461 patients without DM (ICD-10-CM codes: E08, E09, E10, E11, and E13; ICD-9-CM codes: 250). The date of the initial diagnosis of EC was designated as the index date. In our observational cohort, EC cases were matched 1:1 to control subjects based on age, sex, occupation, geographic location, and index year, starting from January 1, 2007, in accordance with the enrollment period in the NTCRD.

### Outcome variables

Both EC patients with or without DM were followed until the incident fracture was identified (osteoporotic fracture, hip fracture, vertebral fracture, upper extremities fracture), withdrawal from the insurance, or the end of 2019. The ICD codes of the fractures were listed in **Supplementary Table S1**. The patients who were newly diagnosed with a fracture at least three times at the outpatient clinic (13) or at least one time during admission (14) between 2007 and 2018 were considered the potential study population. The incidence of fracture was the sum of all events and was calculated per 1,000 person-years.

### Variable definitions

Covariates that might be associated with the development of fracture were included in the analyses. These included age (categorized as 20-39, 40-59, >= 60), occupation (categorized as white collar, blue collar, and other), urbanization (municipality and non-municipality), average monthly income (categorized as <35000 NTD, >= 35000 NTD), baseline comorbidities, radiotherapy, chemotherapy, target therapy, hormone therapy, cancer staging, grade, and the pathological status of the estrogen receptor (ER) and progesterone receptor (PR). For sensitivity analysis, we further evaluated the risk for osteoporosis and osteoporotic fracture in patients with EC within the 2011–2018 cohort. This included BMI as a potential risk covariate. Comorbidities included coronary artery disease (15), stroke, heart failure, chronic kidney disease (CKD) (16), chronic obstructive pulmonary disease (COPD) (17), rheumatologic disease (18), polycystic ovary syndrome (PCOS) (19). The ICD codes of the comorbidities were listed in **Supplementary Table S1**. The medications of hormone therapy included clomifene, raloxifene, bazedoxifene, tamoxifen, gestrinone, progesterone, norethisterone, allylestrenol, medroxyprogesterone, medroxyprogesterone, gestrinone, progesterone, dienogest, hydroxyprogesterone, dydrogesterone, medrogestone, levonorgestrel, ethisterone, letrozole, exemestane, and anastrozole.

### Statistical analysis

The prevalence of demographic characteristics and comorbidities was compared between populations with and without EC using t-tests for continuous variables and Chi-squared tests for categorical ones. Cox proportional hazards analysis was used to estimate hazard ratios (HRs) and 95% confidence intervals (CIs) for fracture and mortality events. Considering the competing death events, we estimated the sub-distribution hazard model to predict the probability of the fracture risk with adjusted multivariable in the competing risks context using Fine and Gray method. Multivariate analysis, including basic demographics, comorbidities, and cancer-related medical records, was performed to calculate adjusted hazard ratios (aHRs). In the fully adjusted model, we adjusted for age, occupation, income, urbanization and comorbidities.

The basic characteristics and prevalence of comorbidities were compared between EC patients with and without DM. The cumulative incidence of fractures was estimated using the Kaplan–Meier method, and the HRs and 95% CIs for fractures were calculated using Cox proportional hazards models during the follow-up period. Demographic variables, comorbidities, cancer characteristics and treatment were included in multivariable models to estimate the aHRs: Model 1 adjusted for age, Model 2 adjusted for Model 1 variable, occupation, income, and urbanization, Model 3 adjusted for Model 2 variables and comorbidities, and Model 4 adjusted for Model 3 variables, cancer characteristics, and treatment. We also used the Bonferroni test to adjust for multiple comparisons.

For sensitivity analysis, demographic variables (including BMI), comorbidities, cancer characteristics and treatment were included in multivariable models to estimate the aHRs: Model 1 adjusted for age, Model 2 adjusted for Model 1 variable, occupation, income, and urbanization, Model 3 adjusted for Model 2 variables, comorbidities and BMI, and Model 4 adjusted for Model 3 variables, cancer characteristics and treatment. Statistical analyses were conducted with SAS 9.4 and STATA version 14 (StataCorp), with significance defined as a two-tailed p-value of < 0.05.

## Results

### Fracture risk among patients with and without EC

We identified 15,505 patients with EC between 2007 and 2018 (**Figure 1**). The competing risk models showed that patients with EC had a no significant association of hip, vertebra, and upper extremity fractures (**Table 1**). When compared with the general population, patients with EC and DM (HR 0.56 [95% CI 0.37–0.85]) or aged older than 65 years (HR 0.61 [95% CI 0.41–0.9]) had a lower risk of hip fracture than the control group (**Supplementary Table S2**). When compared with the general population, the interaction effect on vertebral fracture risk differed within the subgroups of age, DM, and COPD (**Supplementary Table S3**). When compared with the general population, the interaction effect on upper extremity fracture risk differed within the subgroups of age and COPD (**Supplementary Table S4**).

**Table 1:** The fracture event number, follow-up person-year, the incidence rate and the hazard ratio and 95% confidence interval in patients with and without endometrial cancer (competing risk).

Hip fracture					Vertebral fracture					Upper extremity fracture				
Outcome variables	Without endometrial cancer	With endometrial cancer			Outcome variables	Without endometrial cancer	With endometrial cancer			Outcome variables	Without endometrial cancer	With endometrial cancer		
**N**	15497	15497			**N**	15497	15497			**N**	15497	15497		
**Event**	117	105			**Event**	140	121			**Event**	267	275		
**Person-year**	82826.3	75019.53			**Person-year**	82826.31	74976.61			**Person-year**	82425.95	74478.18		
**Incidence rate (/1000person-year)**	1.4	1.4			**Incidence rate (/1000person-year)**	1.7	1.6			**Incidence rate (/1000person-year)**	3.2	3.7		
	**HR**	**HR**	**95% CI**			**HR**	**HR**	**95% CI**			**HR**	**HR**	**95% CI**	
**Model 0**	1	0.90	0.69	1.17	**Model 0**	1	0.87	0.68	1.11	**Model 0**	1	1.04	0.88	1.23
**Model 1**	1	0.90	0.69	1.17	**Model 1**	1	0.87	0.68	1.11	**Model 1**	1	1.04	0.88	1.23
**Model 2**	1	0.91	0.70	1.18	**Model 2**	1	0.87	0.68	1.12	**Model 2**	1	1.03	0.87	1.22
**Model 3**	1	0.77	0.58	1.01	**Model 3**	1	0.77	0.60	1.00	**Model 3**	1	0.96	0.81	1.15

Incidence rate, per 1,000 person-years, HR, hazard ratio; CI, confidence interval.

Model 1: Adjust for age.

Model 2: Adjust for model 1+occupation, income, urbanization.

Model 3: Adjust for model 2+comorbidities.

### Fracture risk among patients with EC with and without DM

**Figure 2:**
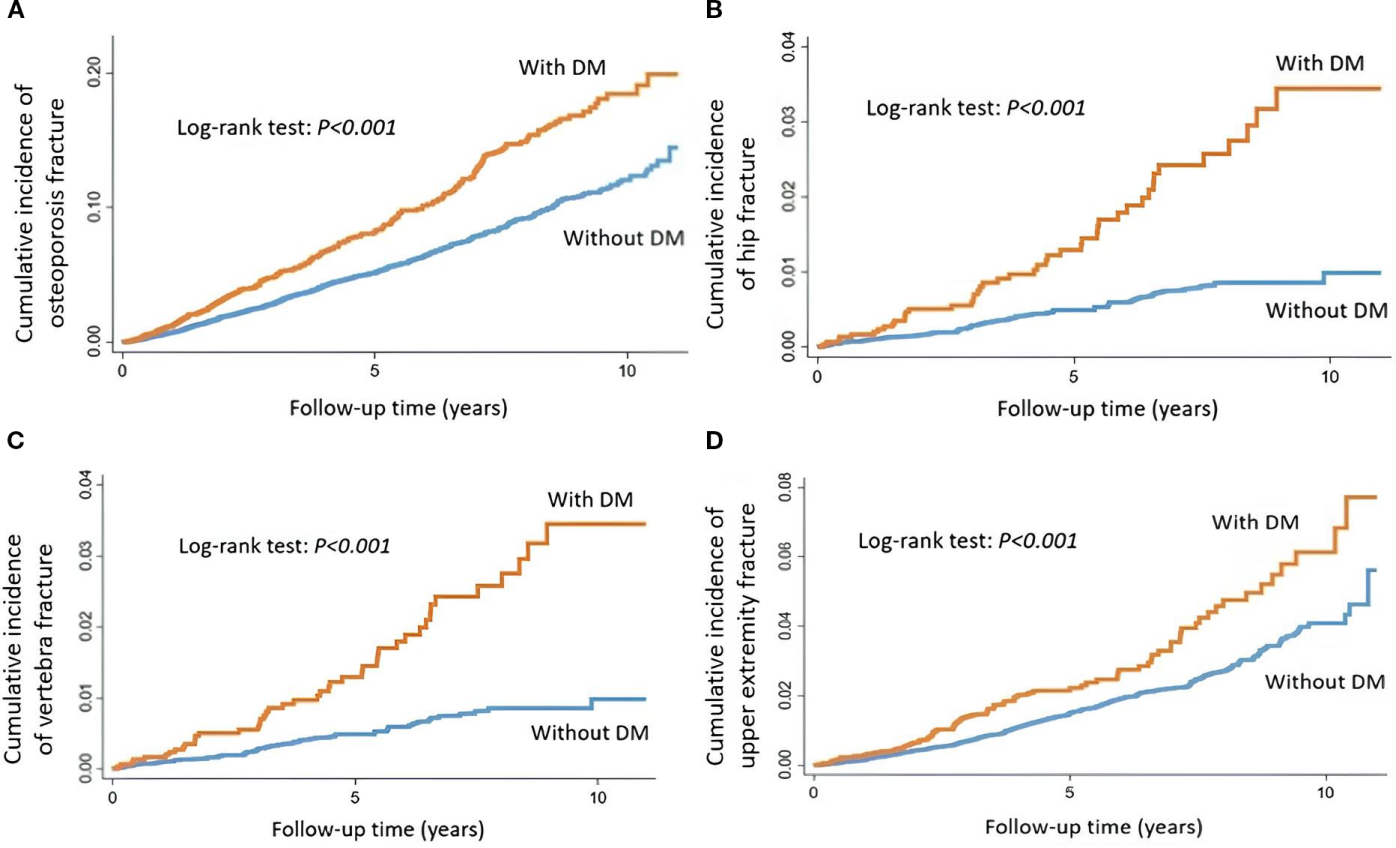
Cumulative incidence of **(A)** osteoporotic fracture, **(B)** hip fracture, **(C)** vertebral fracture, and **(D)** upper extremity fracture in endometrial cancer patients with and without DM.

Of the 15,505 patients with EC from 2007 to 2018, there were 3,044 patients with and 12,461 patients without DM. The mean age of patients with EC, but without DM was 53.6 years. On the other hand, the mean age of patients with EC and DM was 57.5 years (**Table 2**). Patients with EC and DM had a higher prevalence of CVD, CKD, and COPD. These patients also received more RT and chemotherapy than those without DM. Patients with stages 3, 4, and grade 3 had a higher proportion of DM. Patients with DM had more osteoporotic fracture events (8.71%) than those without DM (5.4%). The cumulative incidence of osteoporotic fracture (log-rank test: *P* < 0.001), hip fracture (log-rank test: *P* < 0.001), vertebral fracture (log-rank test: *P* < 0.001), and upper extremity fracture (log-rank test: *P* < 0.001) in patients with EC with and without DM were demonstrated in **Figure 2**. Patients with DM had a significantly higher risk of osteoporotic fracture (HR 1.29 [95% CI 1.08-1.55]) (**Table 3**), hip fracture (HR 2.37 [95% CI 1.44-3.92]), and vertebral fracture (HR 1.71 [95% CI 1.06-2.74]). Patients with DM had a no significant association of upper extremity fracture (HR 1.33 [95% CI 0.95-1.87]) after they were adjusted for comorbidities, cancer characteristics, and treatment. We used the Bonferroni test for adjustment for multiple comparisons, and the results were demonstrated in **Supplementary Table S5**. Factors associated with each kind of fracture in patients with EC and DM were demonstrated in **Table 4**. Only age was demonstrated to have a significant interaction effect on hip fracture. Comorbidities and cancer characteristics seemed to have no interaction effect on fractures.

**Table 2:** Baseline demographic factors and comorbidities of endometrial cancer patients with and without DM (osteoporotic fracture cohort).

Characteristics	Without DM		With DM		p-value
	N=12461		N=3044		
	n	%	n	%	
**Age, years**					<0.001
20-39	1013	8.13	184	6.04	
40-59	8304	66.64	1550	50.92	
≧60	3144	25.23	1310	43.04	
mean (SD)	53.6 (9.91)		57.5 (10.74)		
**Occupation**					<0.001
White collar	1989	15.96	563	18.5	
Blue collar	6648	53.35	1366	44.88	
Other	3824	30.69	1115	36.63	
**Urbanization**					0.006
1	3758	30.16	996	32.72	
2	8703	69.84	2048	67.28	
**Income**					<0.001
<30000	9776	78.45	2543	83.54	
≧30000	2685	21.55	501	16.46	
**Comorbidities**					
CVD	482	3.87	465	15.28	<0.001
CKD	2396	19.23	1915	62.91	<0.001
COPD	2317	18.59	810	26.61	<0.001
Rheumatologic disease	961	7.71	231	7.59	0.819
PCOS	370	2.97	100	3.29	0.362
**Radiotherapy**					<0.001
No	8728	70.04	2016	66.23	
Yes	3733	29.96	1028	33.77	
**Chemotherapy**					0.002
No	9554	76.67	2251	73.95	
Yes	2907	23.33	793	26.05	
**Target therapy**					0.181
No	12407	99.57	3036	99.74	
Yes	54	0.43	8	0.26	
**Hormone therapy**					0.843
No	11618	93.23	2835	93.13	
Yes	843	6.77	209	6.87	
**Staging**					0.031
I	9005	74.5	2173	72.48	
II	720	5.96	185	6.17	
III	1682	13.92	479	15.98	
IV	680	5.63	161	5.37	
**Grade**					<0.001
1 and 2	5346	62.75	1267	58.15	
3	3173	37.25	912	41.85	
**ER**					0.184
negative	3574	29.98	842	28.58	
low	2612	21.91	684	23.22	
strong	5736	48.11	1420	48.2	
**PR**					0.094
negative	4415	37.57	1028	35.4	
low	2810	23.91	721	24.83	
strong	4525	38.51	1155	39.77	
**Osteoporotic fracture**					<0.001
No	11788	94.6	2779	91.29	
Yes	673	5.4	265	8.71	
**Hip fracture**					<0.001
No	12402	99.53	2998	98.49	
Yes	59	0.47	46	1.51	
**Vertebral fracture**					<0.001
No	12387	99.41	2997	98.46	
Yes	74	0.59	47	1.54	
**Upper extremity fracture**					<0.001
No	12260	98.41	2967	97.47	
Yes	198	1.59	77	2.53	

Bold values: the significance was defined as a p-value of < 0.05.

**Table 3:** The fracture event number, follow-up person-year, the incidence rate and the hazard ratio and 95% confidence interval in endometrial cancer patients with and without DM.

Osteoporotic fracture					Hip fracture					Vertebral fracture					Upper extremity fracture				
Outcome variables	Without DM	With DM			Outcome variables	Without DM	With DM			Outcome variables	Without DM	With DM			Outcome variables	Without DM	With DM		
**N**	12461	3044			**N**	12461	3044			**N**	12461	3044			**N**	12461	3044		
**Event**	673	265			**Event**	59	46			**Event**	74	47			**Event**	198	77		
**Person-year**	58641	14061.92			**Person-year**	60391	14645.62			**Person-year**	60352	14641.71			**Person-year**	59968	14527.16		
**Incidence rate (/1000person-year)**	11.5	18.8			**Incidence rate (/1000person-year)**	1	3.1			**Incidence rate (/1000person-year)**	1.2	3.2			**Incidence rate (/1000person-year)**	3.3	5.3		
	HR	HR	95% CI			HR	HR	95% CI			HR	HR	95% CI			HR	HR	95% CI	
**Model 0**	1	1.64	1.4	1.92	**Model 0**	1	3.51	2.27	5.43	**Model 0**	1	2.81	1.87	4.24	**Model 0**	1	1.48	1.1	1.99
**Model 1**	1	1.41	1.2	1.65	**Model 1**	1	2.8	1.8	4.37	**Model 1**	1	2.27	1.5	3.44	**Model 1**	1	1.37	1.02	1.85
**Model 2**	1	1.39	1.18	1.64	**Model 2**	1	2.78	1.78	4.33	**Model 2**	1	2.19	1.44	3.32	**Model 2**	1	1.36	1.01	1.83
**Model 3**	1	1.29	1.07	1.54	**Model 3**	1	2.4	1.45	3.96	**Model 3**	1	1.68	1.05	2.7	**Model 3**	1	1.33	0.94	1.86
**Model 4**	1	1.29	1.08	1.55	**Model 4**	1	2.37	1.44	3.92	**Model 4**	1	1.71	1.06	2.74	**Model 4**	1	1.33	0.95	1.87

Model 1: Adjusted for age.

Model 2: Adjusted for model 1+occupation, income, urbanization.

Model 3: Adjusted for model 2+comorbidities.

Model 4: Adjusted for model 3+cancer characteristics and treatment.

Bold values: the significance was defined as a p-value of < 0.05.

**Table 4:** Subgroup analysis among endometrial cancer patients with and without DM.

Characteritics	Hip fracture				Characteritics	Vertebral fracture				Characteritics	Upper extremity fracture			
	With DM					With DM					With DM			
	HR	95% CI		*P*		HR	95% CI		*P*		HR	95% CI		*P*
**Age, years**				0.011	**Age, years**				0.337	**Age, years**				0.724
<60	2.34	1.08	5.04	0.03		1.74	0.84	3.58	0.134		1.33	0.87	2.03	0.184
≧60	2.49	1.27	4.87	0.01		1.68	0.9	3.14	0.102		1.30	0.74	2.31	0.362
**ER**				0.125	**ER**				0.993	**ER**				0.674
negative	2.72	1.11	6.63	0.028	negative	1.76	0.7	4.42	0.233	negative	1.59	0.8	3.14	0.185
positive	2.26	1.22	4.19	0.01	positive	1.72	0.99	2.97	0.054	positive	1.23	0.83	1.82	0.302
**PR**				0.31	**PR**				0.932	**PR**				0.903
negative	2.44	1.03	5.79	0.043	negative	1.77	0.76	4.14	0.188	negative	1.48	0.79	2.75	0.221
positive	2.34	1.25	4.36	0.008	positive	1.69	0.96	2.98	0.068	positive	1.26	0.84	1.88	0.271
**Staging**				0.748	**Staging**				0.204	**Staging**				0.176
I and II	2.49	1.44	4.3	0.001	I and II	1.61	0.95	2.74	0.078	I and II	1.44	1.01	2.06	0.045
III and IV	2.13	0.64	7.08	0.217	III and IV	2.89	1.03	8.14	0.044	III and IV	0.51	0.17	1.49	0.216
**Radiotherapy**				0.184	**Radiotherapy**				0.333	**Radiotherapy**				0.971
No	3.13	1.7	5.74	<0.001	No	1.49	0.8	2.77	0.21	No	1.48	0.97	2.25	0.07
Yes	1.3	0.53	3.19	0.564	Yes	2.02	0.96	4.24	0.064	Yes	1.07	0.61	1.9	0.811
**Chemotherapy**				0.578	**Chemotherapy**				0.771	**Chemotherapy**				0.1
No	2.33	1.32	4.13	0.004	No	1.54	0.91	2.62	0.109	No	1.44	1	2.08	0.05
Yes	2.59	0.9	7.48	0.078	Yes	2.32	0.82	6.62	0.115	Yes	0.75	0.29	1.96	0.559
**Target therapy**				1	**Target therapy**				1	**Target therapy**				1
No	2.38	1.44	3.93	0.001	No	1.7	1.06	2.73	0.027	No	1.32	0.94	1.86	0.108
Yes					Yes					Yes				
**Hormone therapy**				0.684	**Hormone therapy**				0.988	**Hormone therapy**				0.584
No	2.41	1.44	4.01	0.001	No	1.97	1.21	3.2	0.006	No	1.33	0.93	1.89	0.115
Yes	3.01	0.18	51	0.445	Yes	0	0		0.997	Yes	1.2	0.34	4.25	0.782
**CVD**				0.968	**CVD**				0.724	**CVD**				0.823
No	2.38	1.39	4.1	0.002	No	1.62	0.97	2.72	0.067	No	1.34	0.92	1.94	0.126
Yes	2.77	0.66	11.57	0.163	Yes	2.527	0.68	9.36	0.165	Yes	1.45	0.58	3.64	0.426
**CKD**				0.202	**CKD**				0.623	**CKD**				0.659
No	1.64	0.74	3.64	0.224	No	1.38	0.63	3.04	0.421	No	1.23	0.76	1.99	0.406
Yes	3.41	1.6	7.26	0.002	Yes	1.92	1.04	3.56	0.037	Yes	1.41	0.86	2.31	0.178
**COPD**				0.142	**COPD**				0.691	**COPD**				0.537
No	3.11	1.7	5.7	<0.001	No	1.58	0.87	2.86	0.136	No	1.48	0.97	2.26	0.067
Yes	1.28	0.52	3.14	0.592	Yes	1.94	0.88	4.26	0.098	Yes	1.15	0.66	2.02	0.623
**Rheumatologic disease**				0.137	**Rheumatologic disease**				0.72	**Rheumatologic disease**				0.071
No	2.19	1.29	3.71	0.004	No	1.82	1.09	3.03	0.022	No	1.27	0.88	1.82	0.203
Yes	7.39	1.04	52.26	0.045	Yes	1.16	0.27	4.97	0.846	Yes	1.96	0.71	5.46	0.196
**Polycystic ovary syndrome**				1	**Polycystic ovary syndrome**				1	**Polycystic ovary syndrome**				0.521
No	2.38	1.44	3.93	0.001	No	1.7	1.06	2.73	0.027	No	1.29	0.91	1.82	0.149
Yes					Yes					Yes	4.26	0.13	144.94	0.42

Bold values: the significance was defined as a p-value of < 0.05.

### Sensitivity analysis

As for the 2011–2018 cohort, which added BMI as one of the covariates, baseline demographic factors and comorbidities of EC patients with and without DM were demonstrated in **Supplementary Table S6**. Most of the patients with DM had a BMI ≥25. Patients with EC and DM in the 2011–2018 cohort had a significantly higher incidence of osteoporosis fracture (log-rank test: *P* < 0.001) (**Supplementary Figure S1**). Patients with EC and DM in the 2011–2018 cohort had a significantly higher risk of osteoporotic fracture (HR 1.35 [95% CI 1.09–1.69]) (**Supplementary Table S7**).

## Discussion

Our study showed that patients with EC had a no significant association of hip, vertebral, and upper extremity fractures when compared with the general population. In all patients with EC, those with DM had a significantly higher risk of osteoporotic fracture, hip fracture, and vertebral fracture than those without DM. Patients with DM had a no significant association of upper extremity fracture compared with those with EC but without DM.

The present study demonstrated that patients with EC had a no significant association of hip, vertebra and upper extremity fracture when compared to the general population, which was not consistent with another population-based study of 2111 patients with EC (20), which showed a reduced risk of hip fracture in EC patients. This study had fewer patients than the current study (20), and the patients were followed up starting at age 50 (20). The present study included all patients older than 20 years. The previous study also demonstrated that age was not a determinant of the risk of hip fracture (20). On the other hand, we found that patients with EC and DM or aged older than 65 years had a lower risk of hip fracture than patients without EC. Since DM and older age are both well-known risk factors for fractures (21), lower risk of hip fractures in patients with EC may be attributed to higher BMI in patients with EC in the present study (20). Again, when compared with the general population, the interaction effect on vertebral fracture risk differed within subgroups of age, DM, and COPD. The difference between the fracture risk of the hip and vertebra could be attributed to the early bone loss in the trabecular bone, but with increasing age, the bone loss is restricted to the cortical region (22). Vertebral fractures are more common in patients younger than 65 years, and hip fractures are more frequent in patients older than 65 years (22). Previous studies revealed that patients with DM were vulnerable to postural instability due to altered motion perception (11). In addition, severe hypoglycemia was associated with a greater prevalence of falls (23), which may also put patients with DM at risk of fractures. With respect to COPD, smoking (24), reduced physical activity (25), low body weight (26), sarcopenia (27), systemic inflammation (28), glucocorticoid (29), vitamin D deficiency (30), hypoxemia and hypercapnia (31, 32), and anemia (33) were all associated with a higher risk of fracture. When compared with the general population, the interaction effect on upper extremity fracture risk also differed within subgroups of age and COPD.

Our study indicated that when comparing EC patients with and without DM, only age was demonstrated to have a significant interaction effect on hip fracture. Comorbidities, cancer treatment and characteristics seemed to have no interaction effect on fractures. A previous study involving 40 patients with cervical cancer revealed that changes in BMD of the lumbar spine after pelvic RT were not significant (34). Nonetheless, another study with 557 patients with cervical cancer showed that those with higher age, lower body weight, and higher radiation dose may suffer from pelvic fracture (35). In addition, another study of 239 patients, of whom 73 patients were diagnosed with EC, found a significant decrease in BMD after pelvic RT (36). As observed in our study, since patients with DM were diagnosed with more advanced EC, they received more RT and chemotherapy. Those who did not receive adjuvant therapy may suffer more from other comorbidities that restrain them from receiving further treatment. The staging and grade of EC and the diagnosis of DM were not analyzed in previous studies (34–36). Our study showed that cancer staging and grade had no interaction effect on hip, vertebral, and upper extremity fractures. Previous research demonstrated that higher BMI, compared to lower individuals, had a protective effect against fracture incidence (20). In our sensitivity analysis, which added BMI as one of the covariates, patients with EC and DM still demonstrated a significantly higher risk of osteoporotic fracture than patients without DM.

The strength of this study lies in the fact that it uses the largest number of patients’ data to date. The study focused on the fracture risk of EC patients with or without DM. Longer follow-up periods strengthened our evidence compared to previous investigations. Additionally, we conducted subgroup analyses to account for potential confounders, further enhancing the robustness of our findings. There are several limitations acknowledged within the research. First, rather than a randomized control design, the present observational study lacked the sufficient causality between the fracture risk and EC patients with DM. However, the nationally population-based cohort study still demonstrated significant evidence. Second, we could not calculate the window of time in which the 3 outpatient fractures occurred, nor could we determine whether the patients had similar fracture sites or not. Third, hormone therapy in this study included bone-positive and bone-negative effect, which may misestimate the interaction effect of hormone therapy on fracture. Fourth, we only excluded patients with history of osteoporotic fractures because diagnosis of osteoporosis and use of osteoporosis medication may be underestimated by NHIRD. Fifth, there might be persistent unmeasured covariates, particularly concerning cancer characteristics with potential interactions, collinearity, or dependencies among variables related to cancer and its treatment (e.g., estrogen level, ER and PR status, staging, radiotherapy, chemotherapy, targeted therapy, and hormone therapy). Although the basic demographic characteristic and detailed cancer information, such as staging and treatment, were all adjusted in our full model, future studies can benefit from addressing these dependencies through more advanced statistical techniques or alternative study designs, such as calculating variance inflation factors (VIFs), examining correlation matrices, stratification on cancer staging or investigating interaction terms of hormone receptor status which may potential confound the fracture risk.

## Conclusion

According to our study, patients with EC had a no significant association of fracture, while DM increased the fracture risk in patients with EC. Age had a significant interaction effect on fracture. Since there were several limitations in the present study, such as lacking more detailed treatment and cancer staging classification, the results should be interpreted cautiously and more comprehensive studies are still required.

## Data Availability

The original contributions presented in the study are included in the article/**Supplementary Material**. Further inquiries can be directed to the corresponding author.
