# Supplementary material for: Association between endometrial cancer and subsequent risk of fracture: a national cohort study
Source: Front Endocrinol (Lausanne). 2025 Oct 7;16:1570426. doi: 10.3389/fendo.2025.1570426 (PMC12537369; doi:10.3389/fendo.2025.1570426)
Supplement: Supplementary file 1 [file DataSheet1.doc]

**Supplemental Table 1** ICD codes

| Disease | ICD-10 | ICD-9 |
| --- | --- | --- |
| osteoporotic fracture | M80.0 | 733.10 |
| hip fracture | S72 | 820-821 |
| vertebral fracture | M80.08, S32.0, S22.0-S22.089, T08 | 809 |
| upper extremities fracture | S52.0-S52.6, S62.0, M80.01-M80.039, S42.20-S42.40, T10 | 812-814 |
| coronary artery disease | I20, I21, I22, I23, I24, I25 | 410, 411, 412, 413, 414, 429 |
| stroke | I60, I61, I62, I63 | 430, 431, 432, 433 |
| heart failure | I50 | 428 |
| chronic kidney disease | N18, N19 | 585, 586, 593 |
| chronic obstructive pulmonary disease | J44, J45, J47 | 491, 493, 494, 496 |
| rheumatologic disease | L93, L94 | 695, 701 |
| polycystic ovary syndrome | E28.2 | 256.4 |

**Supplemental Table** **2** Subgroup analysis among patients with and without endometrial cancer: hip fracture

|  | **with endometrial cancer** | | | |
| --- | --- | --- | --- | --- |
|  | **HR** | **95% CI** | | ***P*** |
| **Overall** |  |  |  |  |
| **Age, years** |  |  |  | <0.001 |
| <65 | 1.24 | 0.85 | 1.82 | 0.268 |
| ≧65 | 0.61 | 0.41 | 0.9 | 0.013 |
| **CVD** |  |  |  | 0.465 |
| No | 0.93 | 0.68 | 1.26 | 0.638 |
| Yes | 0.63 | 0.35 | 1.16 | 0.136 |
| **CKD** |  |  |  | 0.564 |
| No | 0.96 | 0.67 | 1.38 | 0.817 |
| Yes | 0.76 | 0.51 | 1.14 | 0.182 |
| **DM** |  |  |  | 0.02 |
| No | 1.1 | 0.77 | 1.55 | 0.608 |
| Yes | 0.56 | 0.37 | 0.85 | 0.006 |
| **COPD** |  |  |  | 0.618 |
| No | 1 | 0.71 | 1.41 | 0.987 |
| Yes | 0.67 | 0.43 | 1.06 | 0.087 |
| **Rheumatologic disease** |  |  |  | 0.656 |
| No | 0.9 | 0.68 | 1.2 | 0.486 |
| Yes | 0.75 | 0.31 | 1.79 | 0.514 |
| **Polycystic ovary syndrome** |  |  |  | 0.967 |
| No | 0.88 | 0.67 | 1.15 | 0.346 |
| Yes |  |  |  |  |

**Supplemental Table 3** Subgroup analysis among patients with and without endometrial cancer: vertebral fracture

|  | **with endometrial cancer** | | | |
| --- | --- | --- | --- | --- |
|  | **HR** | **95% CI** | | ***P*** |
| **Overall** |  |  |  |  |
| **Age, years** |  |  |  | <0.001 |
| <65 | 0.8 | 0.59 | 1.09 | 0.161 |
| ≧65 | 0.98 | 0.64 | 1.49 | 0.913 |
| **CVD** |  |  |  | 0.373 |
| No | 0.81 | 0.62 | 1.06 | 0.129 |
| Yes | 1.39 | 0.67 | 2.86 | 0.374 |
| **CKD** |  |  |  | 0.124 |
| No | 0.88 | 0.64 | 1.22 | 0.442 |
| Yes | 0.83 | 0.56 | 1.23 | 0.347 |
| **DM** |  |  |  | 0.019 |
| No | 0.89 | 0.66 | 1.2 | 0.434 |
| Yes | 0.8 | 0.51 | 1.25 | 0.323 |
| **COPD** |  |  |  | 0.016 |
| No | 0.81 | 0.6 | 1.11 | 0.191 |
| Yes | 0.97 | 0.63 | 1.48 | 0.87 |
| **Rheumatologic disease** |  |  |  | 0.338 |
| No | 0.9 | 0.69 | 1.17 | 0.43 |
| Yes | 0.72 | 0.36 | 1.45 | 0.363 |
| **Polycystic ovary syndrome** |  |  |  | 0.96 |
| No | 0.88 | 0.69 | 1.14 | 0.332 |
| Yes | 0 | 0 |  | 1 |

**Supplemental Table 4** Subgroup analysis among patients with and without endometrial cancer: upper extremity fracture

|  | **with endometrial cancer** | | | |
| --- | --- | --- | --- | --- |
|  | **HR** | **95% CI** | | ***P*** |
| **Overall** |  |  |  |  |
| **Age, years** |  |  |  | 0.038 |
| <65 | 1.1 | 0.91 | 1.33 | 0.342 |
| ≧65 | 0.95 | 0.63 | 1.42 | 0.792 |
| **CVD** |  |  |  | 0.198 |
| No | 1.04 | 0.87 | 1.25 | 0.672 |
| Yes | 1.76 | 0.98 | 3.17 | 0.061 |
| **CKD** |  |  |  | 0.151 |
| No | 1.18 | 0.96 | 1.45 | 0.108 |
| Yes | 0.87 | 0.63 | 1.21 | 0.415 |
| **DM** |  |  |  | 0.063 |
| No | 1.22 | 1 | 1.48 | 0.049 |
| Yes | 0.73 | 0.51 | 1.04 | 0.081 |
| **COPD** |  |  |  | <0.001 |
| No | 0.99 | 0.81 | 1.22 | 0.942 |
| Yes | 1.32 | 0.97 | 1.81 | 0.081 |
| **Rheumatologic disease** |  |  |  | 0.76 |
| No | 1.12 | 0.93 | 1.34 | 0.231 |
| Yes | 0.85 | 0.49 | 1.47 | 0.548 |
| **Polycystic ovary syndrome** |  |  |  | 0.857 |
| No | 1.09 | 0.91 | 1.29 | 0.352 |
| Yes | 0.91 | 0.08 | 10.18 | 0.94 |

**Supplemental Table 5** The fracture event number, follow-up person-year, the incidence rate and the hazard ratio and 95% confidence interval in endometrial cancer patients with and without DM (adjustment for multiple comparisons: The Bonferroni test, adjusted p value to 0.05/4 = 0.013)

| **Osteoporotic fracture** | | | | | | **Hip fracture** | | | | | | | | | **Vertebral fracture** | | | | | | | | | | **Upper extremity fracture** | | | | | | | |  |
| --- | --- | --- | --- | --- | --- | --- | --- | --- | --- | --- | --- | --- | --- | --- | --- | --- | --- | --- | --- | --- | --- | --- | --- | --- | --- | --- | --- | --- | --- | --- | --- | --- | --- |
|  | **without DM** | **with DM** | | | |  | | **without DM** | | **with DM** | | | | |  | | **without DM** | | **with DM** | | | | | |  | **without DM** | | **with DM** | | | | |  |
|  | HR | HR | 95% CI | | ***P*** | |  | | HR | | HR | 95% CI | | ***P*** | |  | | HR | | HR | 95% CI | | ***P*** |  | | | HR | | HR | 95% CI | | ***P*** | |
| **Model 0** | 1 | 1.64 | 1.4 | 1.92 | <0.01 | | **Model 0** | | 1 | | 3.51 | 2.27 | 5.43 | <0.01 | | **Model 0** | | 1 | | 2.81 | 1.87 | 4.24 | <0.01 | **Model 0** | | | 1 | | 1.48 | 1.1 | 1.99 | 0.01 | |
| **Model 1** | 1 | 1.41 | 1.2 | 1.65 | <0.01 | | **Model 1** | | 1 | | 2.8 | 1.8 | 4.37 | <0.01 | | **Model 1** | | 1 | | 2.27 | 1.5 | 3.44 | <0.01 | **Model 1** | | | 1 | | 1.37 | 1.02 | 1.85 | 0.04 | |
| **Model 2** | 1 | 1.39 | 1.18 | 1.64 | <0.01 | | **Model 2** | | 1 | | 2.78 | 1.78 | 4.33 | <0.01 | | **Model 2** | | 1 | | 2.19 | 1.44 | 3.32 | <0.01 | **Model 2** | | | 1 | | 1.36 | 1.01 | 1.83 | 0.05 | |
| **Model 3** | 1 | 1.29 | 1.07 | 1.54 | <0.01 | | **Model 3** | | 1 | | 2.4 | 1.45 | 3.96 | <0.01 | | **Model 3** | | 1 | | 1.68 | 1.05 | 2.7 | 0.03 | **Model 3** | | | 1 | | 1.33 | 0.94 | 1.86 | 0.1 | |
| **Model 4** | 1 | 1.29 | 1.08 | 1.55 | <0.01 | | **Model 4** | | 1 | | 2.37 | 1.44 | 3.92 | <0.01 | | **Model 4** | | 1 | | 1.71 | 1.06 | 2.74 | 0.03 | **Model 4** | | | 1 | | 1.33 | 0.95 | 1.87 | 0.1 | |

Model 1: Adjusted for age

Model 2: Adjusted for model 1+occupation, income, urbanization

Model 3: Adjusted for model 2+comorbidities

Model 4: Adjusted for model 3+cancer characteristics and treatment

**Supplemental Table 6** Baseline demographic factors and comorbidities of endometrial cancer patients with and without DM (2011-2018 Osteoporotic fracture cohort)

|  | **Without DM** | | **With DM** | |  |
| --- | --- | --- | --- | --- | --- |
|  | **N=10655** | | **N=2554** | |  |
|  | **n** | **%** | **n** | **%** | **p-value** |
| **Age, years** |  |  |  |  | <0.001 |
| 20-40 | 851 | 7.99 | 153 | 5.99 |  |
| 40-60 | 7013 | 65.82 | 1275 | 49.92 |  |
| ≧60 | 2791 | 26.19 | 1126 | 44.09 |  |
| mean (SD) |  |  |  |  |  |
| **Occupation** |  |  |  |  | <0.001 |
| White collar | 1666 | 15.64 | 467 | 18.29 |  |
| Blue collar | 5767 | 54.12 | 1156 | 45.26 |  |
| Other | 3222 | 30.24 | 931 | 36.45 |  |
| **Urbanization** |  |  |  |  | 0.127 |
| 1 | 2767 | 25.97 | 701 | 27.45 |  |
| 2 | 7888 | 74.03 | 1853 | 72.55 |  |
| **Income** |  |  |  |  | <0.001 |
| <30000 | 8352 | 78.39 | 2141 | 83.83 |  |
| ≧30000 | 2303 | 21.61 | 413 | 16.17 |  |
| **Comorbidities** |  |  |  |  |  |
| CVD | 404 | 3.79 | 379 | 14.84 | <0.001 |
| CKD | 1992 | 18.7 | 1595 | 62.45 | <0.001 |
| COPD | 1955 | 18.35 | 682 | 26.7 | <0.001 |
| Rheumatologic disease | 841 | 7.89 | 189 | 7.4 | 0.404 |
| PCOS | 327 | 3.07 | 90 | 3.52 | 0.238 |
| **BMI** |  |  |  |  | <0.001 |
| <18.5 | 309 | 2.98 | 22 | 0.88 |  |
| 18.5 to <25 | 5150 | 49.7 | 600 | 24.07 |  |
| 25 to <30 | 3110 | 30.01 | 932 | 37.38 |  |
| ≥ 30 | 1794 | 17.31 | 939 | 37.67 |  |
| **Radiotherapy** |  |  |  |  | <0.001 |
| No | 7490 | 70.3 | 1690 | 66.17 |  |
| Yes | 3165 | 29.7 | 864 | 33.83 |  |
| **Chemotherapy** |  |  |  |  | 0.002 |
| No | 8137 | 76.37 | 1875 | 73.41 |  |
| Yes | 2518 | 23.63 | 679 | 26.59 |  |
| **Target therapy** |  |  |  |  | 0.199 |
| No | 10601 | 99.49 | 2546 | 99.69 |  |
| Yes | 54 | 0.51 | 8 | 0.31 |  |
| **Hormone therapy** |  |  |  |  | 0.512 |
| No | 9959 | 93.47 | 2378 | 93.11 |  |
| Yes | 696 | 6.53 | 176 | 6.89 |  |
| **Staging** |  |  |  |  | 0.053 |
| I | 7746 | 74.7 | 1840 | 73.02 |  |
| II | 598 | 5.77 | 145 | 5.75 |  |
| III | 1436 | 13.85 | 402 | 15.95 |  |
| IV | 590 | 5.69 | 133 | 5.28 |  |
| **Grade** |  |  |  |  | <0.001 |
| 1 and 2 | 4268 | 61.77 | 1004 | 57.11 |  |
| 3 | 2642 | 38.23 | 754 | 42.89 |  |
| **ER** |  |  |  |  | 0.578 |
| negative | 3574 | 35.33 | 842 | 34.28 |  |
| low | 809 | 8 | 194 | 7.9 |  |
| strong | 5733 | 56.67 | 1420 | 57.82 |  |
| **PR** |  |  |  |  | 0.109 |
| negative | 4415 | 44.4 | 1028 | 42.58 |  |
| low | 1007 | 10.13 | 231 | 9.57 |  |
| strong | 4522 | 45.47 | 1155 | 47.85 |  |
| **Osteoporotic fracture** |  |  |  |  | <0.001 |
| No | 10155 | 95.31 | 2355 | 92.21 |  |
| Yes | 500 | 4.69 | 199 | 7.79 |  |

**Supplemental Figure 1** Cumulative incidence of osteoporotic fracture in endometrial cancer patients with and without DM (2011-2018 Osteoporotic fracture cohort)

**Supplemental Table 7** The fracture event number, follow-up person-year, the incidence rate and the hazard ratio and 95% confidence interval in endometrial cancer patients with and without DM (2011-2018 Osteoporotic fracture cohort)

|  | **without DM** | **with DM** | | |
| --- | --- | --- | --- | --- |
| **N** | 10655 | 2554 | | |
| **Event** | 500 | 199 | | |
| **Person-year** | 43649.72 | 10326.23 | | |
| **Incidence rate (/1000person-year)** | 11.5 | 19.3 | | |
|  | HR | HR | 95% CI | |
| **Model 0** | 1.00 | 1.65 | 1.36 | 2 |
| **Model 1** | 1.00 | 1.44 | 1.18 | 1.74 |
| **Model 2** | 1.00 | 1.42 | 1.17 | 1.72 |
| **Model 3** | 1.00 | 1.37 | 1.10 | 1.70 |
| **Model 4** | 1.00 | 1.35 | 1.09 | 1.69 |
| Model 1: Adjusted for age | | | | |
| Model 2: Adjusted for model 1+occupation, income, urbanization | | | | |
| Model 3: Adjusted for model 2+comorbidities+BMI | | | | |
| Model 4: Adjusted for model 2+comorbidities+cancer characteristics and treatment | | | | |
