# Supplementary figures and images for: Association between endometrial cancer and subsequent risk of fracture: a national cohort study
Source: Front Endocrinol (Lausanne). 2025 Oct 7;16:1570426. doi: 10.3389/fendo.2025.1570426 (PMC12537369; doi:10.3389/fendo.2025.1570426)

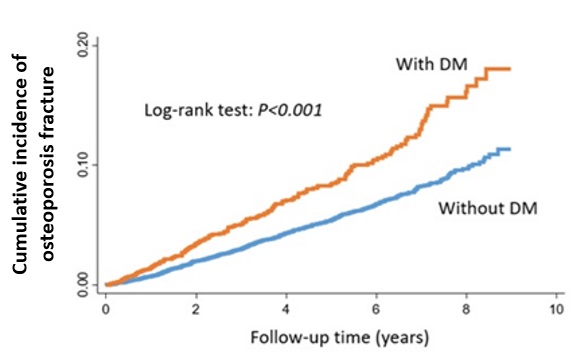

Supplement: Supplementary file 2 [file Image1.jpg]
